# Expression of a homologue of a vertebrate non-visual opsin Opn3 in the insect photoreceptors

**DOI:** 10.1098/rstb.2021.0274

**Published:** 2022-10-24

**Authors:** Mitsumasa Koyanagi, Hayato Honda, Hirohisa Yokono, Ryu Sato, Takashi Nagata, Akihisa Terakita

**Affiliations:** ^1^ Department of Biology, Graduate School of Science, Osaka Metropolitan University, 3-3-138 Sugimoto, Sumiyoshi-ku, Osaka 558-8585, Japan; ^2^ Department of Biology and Geosciences, Graduate School of Science, Osaka City University, 3-3-138 Sugimoto, Sumiyoshi-ku, Osaka 558-8585, Japan; ^3^ The OCU Advanced Research Institute for Natural Science and Technology, Osaka City University, 3-3-138 Sugimoto, Sumiyoshi-ku, Osaka 558-8585, Japan; ^4^ Department of Earth and Space Science, Graduate School of Science, Osaka University, 1-1 Machikaneyama-cho, Toyonaka, Osaka 560-0043, Japan

**Keywords:** rhodopsin, G protein, signal transduction, vision, mosquito, ommatidium

## Abstract

Insect vision starts with light absorption by visual pigments based on opsins that drive Gq-type G protein-mediated phototransduction. Since *Drosophila*, the most studied insect in vision research, has only Gq-coupled opsins, the Gq-mediated phototransduction has been solely focused on insect vision for decades. However, genome projects on mosquitos uncovered non-canonical insect opsin genes, members of the Opn3 or c-opsin group composed of vertebrate and invertebrate non-visual opsins. Here, we report that a homologue of Opn3, MosOpn3 (Asop12) is expressed in eyes of a mosquito *Anopheles stephensi*. *In situ* hybridization analysis revealed that MosOpn3 is expressed in dorsal and ventral ommatidia, in which only R7 photoreceptor cells express MosOpn3. We also found that Asop9, a Gq-coupled visual opsin, exhibited co-localization with MosOpn3. Spectroscopic analysis revealed that Asop9 forms a blue-sensitive opsin-based pigment. Thus, the Gi/Go-coupled opsin MosOpn3, which forms a green-sensitive pigment, is co-localized with Asop9, a Gq-coupled opsin that forms a blue-sensitive visual pigment. Since these two opsin-based pigments trigger different phototransduction cascades, the R7 photoreceptors could generate complex photoresponses to blue to green light.

This article is part of the theme issue ‘Understanding colour vision: molecular, physiological, neuronal and behavioural studies in arthropods’.

## 1. Introduction

Insects are some of the most vision-dependent animals, and numerous studies on vision from molecular to ecological levels have been conducted to uncover its molecular basis, cellular and neural responses, physiological roles and evolution [1,2]. It is well known that opsins are photoreceptor proteins that form photosensitive pigments by binding the chromophore retinal—basically 11-*cis* retinal—as the basis of visual photoreception in insects as well as in other animals, including vertebrates. Opsins are members of G protein-coupled receptors (GPCRs) and act as light-sensitive GPCRs. Thousands of opsins have been identified from various animals, including insects, and are phylogenetically and functionally classified into several groups, many of which are characterized by G protein (*α* subunit) selectivity, such as Gt-, Gq-, Go-, Gs-couped opsins, and so on [3,4]. Activation of each G protein *α* subunit triggers specific signal transduction cascades to produce cellular responses. Interestingly, phototransductions initiated by visual opsins vary between animals. Arthropods including insects employ Gq-coupled opsins for vision [5–9], whereas vertebrates and jellyfish employ Gt-coupled and Gs-couped opsins, respectively [10–13]. Although many animals possess opsins belonging to multiple opsin groups, the fruit fly *Drosophila melanogaster*, the most studied insect in vision research, has seven opsins and they all belong to the Gq-coupled opsin group. This has resulted in Gq-mediated phototransduction being the sole focus of insect visual phototransduction research for decades [14]. However, the genome project researching the malaria mosquito *Anopheles gambiae* found that *Anopheles* has 12 opsins [15]: 10 opsins (Agop1–Agop10) belong to the Gq-coupled opsin group and two opsins (Agop11 and Agop12) belong to the Opn3 or c-opsin group composed of vertebrate and annelid non-visual opsins [16–20]. We have investigated the molecular properties of the Asop12, a homologue of Opn3 from another malaria mosquito *Anopheles stephensi* (MosOpn3), using spectroscopic and biochemical analysis of the recombinant photopigment to reveal that MosOpn3 forms a green-sensitive Gi/Go-coupled opsin-based pigment [21]. Remarkably, MosOpn3 formed photopigments by binding the 13-*cis* form as well as the 11-*cis* form of A1 retinal. Since 13-*cis* retinal is thermally generated from all-*trans* retinal, a retinal isomer present in every tissue, the 13-*cis* retinal-binding property allows for the photopigment formation of MosOpn3 in ‘non-photoreceptive’ tissues [21]. In fact, we have shown by RT-PCR that MosOpn3 is expressed in the body, suggesting the presence of MosOpn3 in extraocular tissues. The analysis also demonstrated that MosOpn3 is expressed in the head. Here, we investigated in detail the expression of MosOpn3 in the eyes of the mosquito *A. stephensi* to reveal that MosOpn3 is expressed specifically in a single kind of photoreceptor R7 of the dorsal and ventral ommatidia, where Asop9, which forms a blue-sensitive Gq-coupled visual pigment, is also expressed. These findings could suggest that this mosquito species employs two types of phototransduction cascades mediated by Gq and Gi/Go in R7, which possibly could affect *Anopheles* colour vision.

## 2. Material and methods

### (a) Animals

The mosquitoes (*Anopheles stephensi*) were reared from eggs kindly provided by Hirotaka Kanuka (Jikei University School of Medicine, Tokyo) and maintained according to the protocol [22].

### (b) cDNA cloning

Partial cDNAs of opsins (Asop1, Asop8 and Asop9) of *A. stephensi* were obtained from RNA isolated from the head by RT-PCR. The primers used for PCR amplification were designed based on the gene sequences found in the genome databases. The full-length cDNAs of the opsins were obtained by using the 3′ RACE and 5′ RACE systems (Invitrogen) as described previously [23].

### (c) Phylogenetic tree inference

The multiple alignment of the amino acid sequences of opsins was performed with the aid of XCED software [24]. Phylogenetic tree inference was performed as described [25]. Briefly, the phylogenetic tree was inferred by the neighbour joining method, and bootstrap analysis was carried out for statistical evaluation. The INSDC accession numbers of the sequences used for analysis are as follows: *Hasarius adansoni* Rh1, AB251846; *Hasarius adansoni* Rh2, AB251847; *Drosophila melanogaster* ninaE, K02315; *Drosophila melanogaster* Rh2, M12896; *Drosophila melanogaster* Rh6, Z86118; *Anopheles stephensi* Asop5, XM_036063766; *Anopheles stephensi* Asop1, LC710138; *Anopheles stephensi* Asop6, XM_036063767; *Anopheles stephensi* Asop7, XM_036047970; *Hasarius adansoni* Rh3, AB251848; *Hasarius adansoni* Rh4, AB506462; *Drosophila melanogaster* Rh3, Y00043; *Drosophila melanogaster* Rh4, M17730; *Anopheles stephensi* Asop8, LC710139; *Drosophila melanogaster* Rh5, U67905; *Anopheles stephensi* Asop9, LC710140; *Drosophila melanogaster* Rh7, AAF49949; *Anopheles stephensi* Asop10, XM_036056155; *Branchiostoma belcheri* melanopsin, AB205400; *Homo sapiens* Opn4 (melanopsin), AF147788; *Homo sapiens* Opn3 (encephalopsin), NM_014322; *Branchiostoma belcheri* Ampihop4, AB050608; *Platynereis dumerilii* ciliary opsin, AY692353; *Anopheles stephensi* MosOpn3 (Asop12), AB753162; *Anopheles stephensi* Asop11, XM_036048022; *Papilio xuthus* Opn3.2, KPI90913; *Papilio xuthus* Opn3.1, KPI90912; *Sympetrum frequens* pteropsin, LC009057; *Photinus pyralis* Opn3, XM_031484106; *Tribolium castaneum* c-opsin, NM_001145478; *Halyomorpha halys* Opn3, XM_014428976; *Apis cerana* pteropsin, XM_017061054; *Aphis gossypii* Opn3, XM_027982987; *Homo sapiens* red, AH005298; *Homo sapiens* green, AH005296; *Homo sapiens* blue, AH003620; *Homo sapiens* rhodopsin, U49742.

### (d) *In situ* hybridization

Preparation of the RNA probes and *in situ* hybridization were carried out as previously described [26]. Briefly, digoxigenin (DIG)- and biotin-labelled antisense and sense RNA probes for *A. stephensi* MosOpn3 (Asop12), Asop1, Asop8 and Asop9 mRNAs were synthesized using the DIG RNA-labelling kit and Biotin RNA-labelling kit (Roche), respectively. In double *in situ* hybridization, DIG-labelled probes for MosOpn3 and biotin-labelled probes for Asop1, Asop8 or Asop9 were used. The mosquito heads were fixed in 4% paraformaldehyde, cryoprotected in 0.1 M phosphate buffer containing 30% sucrose, frozen with OCT Compound (Sakura Finetechnical) and sectioned at 12 µm. DIG-labelled probes were visualized with an alkaline phosphatase-conjugated anti-DIG antibody (Roche), followed by a blue 5-bromo-4-chloro-3-indolyl phosphate/nitro blue tetrazolium colour reaction. Biotin-labelled probes were visualized with the TSA system (Perkin Elmer), followed by horseradish peroxidase diaminobenzidine reaction. Cell nuclei were stained with Hoechst.

### (e) Expression of the opsin-based pigment and spectroscopy

The cDNA of Asop9 was tagged with the monoclonal antibody rho 1D4 epitope sequence (ETSQVAPA). The tagged cDNA was inserted into expression vectors, pcDNA3.1 (Invitrogen) and pUSRα [27], and the recombinant protein was expressed in HEK293S cells. The Asop9-based pigments were reconstituted with 11-*cis* retinal (A1 retinal) as the standard method and the pigments were purified as described [28,29]. Note that the chromophore in *A. stephensi* is not determined but similarity of the absorption maximum wavelength between opsin-based pigments bearing A1 retinal and 11-*cis* 3-hydroxyretinal (A3 retinal), the chromophore in many dipterans, has been demonstrated theoretically and experimentally in a butterfly [30,31]. The absorption spectra of the pigment were recorded at 4°C by using spectrophotometers (UV2450; Shimadzu, Japan, V-750 UV-VIS Spectrophotometer; JASCO International, Japan). Blue and yellow lights were supplied by a 1 kW halogen lamp (Philips) with a 420 nm interference filter and a Y50 glass cutoff filter (Toshiba), respectively.

## 3. Results

### (a) MosOpn3 expression in the mosquito eye

**Figure 1.**
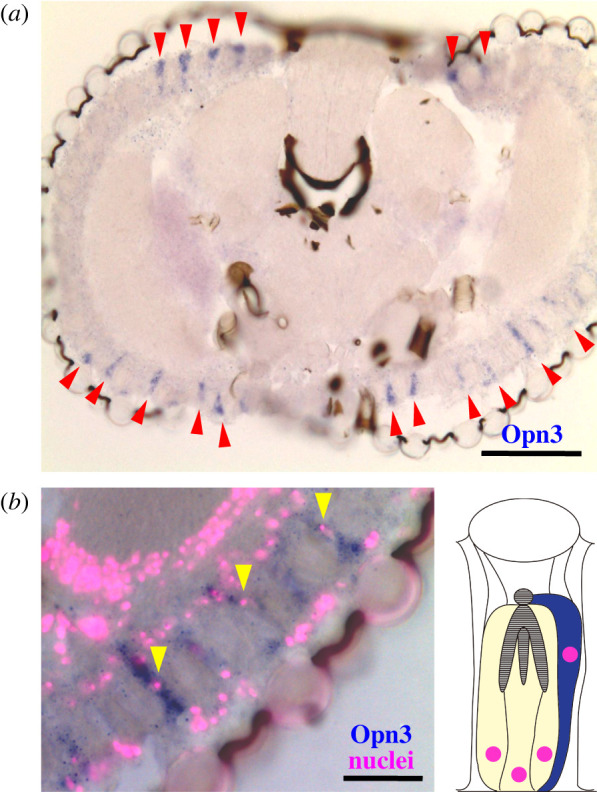
The expression pattern of the Opn3 homologue, MosOpn3 in the *A. stephensi* eye. (*a*) *In situ* hybridization analysis of MosOpn3 (Asop12) in the mosquito eye. The signals derived from MosOpn3 mRNA (arrowheads) were detected in a specific kind of photoreceptor cells in dorsal and ventral ommatidia. (*b*) Visualization of cell nuclei using the Hoechst stain (magenta) in the eye, in which MosOpn3-expressing cells were stained by *in situ* hybridization. The locations of MosOpn3-expressing cell nuclei (arrowheads) indicated that MosOpn3 is expressed in R7. The schematic drawing of mosquito ommatidium is also shown based on the previous report [32]. The lens is illustrated at the top of the ommatidium. R7 (blue) and other photoreceptor cells (yellow) with nuclei (magenta) are shown. Rhabdomeres were also indicated (grey). The scale bars represent 100 μm in (*a*) and 30 μm in (*b*).

In the malaria mosquito (*Anopheles stephensi*), we have previously shown the expression of MosOpn3 (Asop12) in the head and body by RT-PCR [21]. Then, we investigated detailed expression patterns of MosOpn3 in the head of the mosquito by *in situ* hybridization analysis. As a result, the expression of MosOpn3 mRNA was specifically detected in ommatidia located in dorsal and ventral areas of the compound eye in the frontal section (figure 1*a*). In the ommatidia, MosOpn3 expression was observed only in a specific photoreceptor cell. The mosquito ommatidium is composed of eight photoreceptor cells, R1–R8, and they are assembled in a specific pattern; R8 is surrounded by R1 to R6, and R7 is located outside the Rh1–Rh6 ring [32,33]. Among them, R7 is distinguishable from other photoreceptor cells by the unique location of its nucleus, which is isolated from those of R1–6 at the bottom of the ommatidium [32]. Visualization of cell nuclei revealed two photoreceptor nuclear layers, one near the lens and the other near the bottom of the ommatidium (figure 1*b*). Nuclei of MosOpn3-expressing photoreceptor cells are located between the two nuclear layers, which matches one of the criteria of R7. These observations indicated that MosOpn3 is specifically expressed in R7 in dorsal and ventral ommatidia in the mosquito.

### (b) Co-localization of MosOpn3 and a canonical insect visual opsin in photoreceptor cells

**Figure 2.**
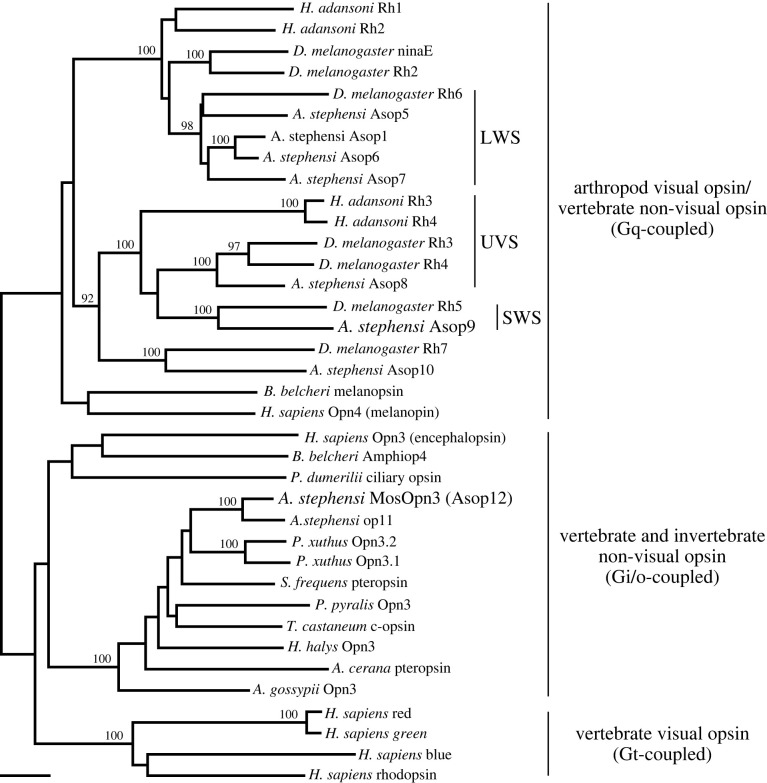
The phylogenetic relationship of insect opsins. The composite tree of the Gq-coupled opsin, the Opn3 and the Gt-coupled opsin groups was inferred with the NJ method. In addition to virtually all opsins of *A. stephensi* and *D. melanogaster*, several opsins representing each group are included. For the Opn3 group, representatives of the Opn3 homologues of insects also called c-opsin or pteropsin are included to show their wide distribution in insects. Insect Gq-coupled visual opsins are classified based on the spectral sensitivity, as shown on the right of the tree. LWS, UVS and SWS indicate the subgroups of LWS, UVS and SWS opsins, respectively. The MosOpn3 and Asop9 are highlighted with bold text. Bootstrap probabilities greater than 90% are indicated at each branch node. The scale bar represents 0.1 substitutions per site.

**Figure 3.**
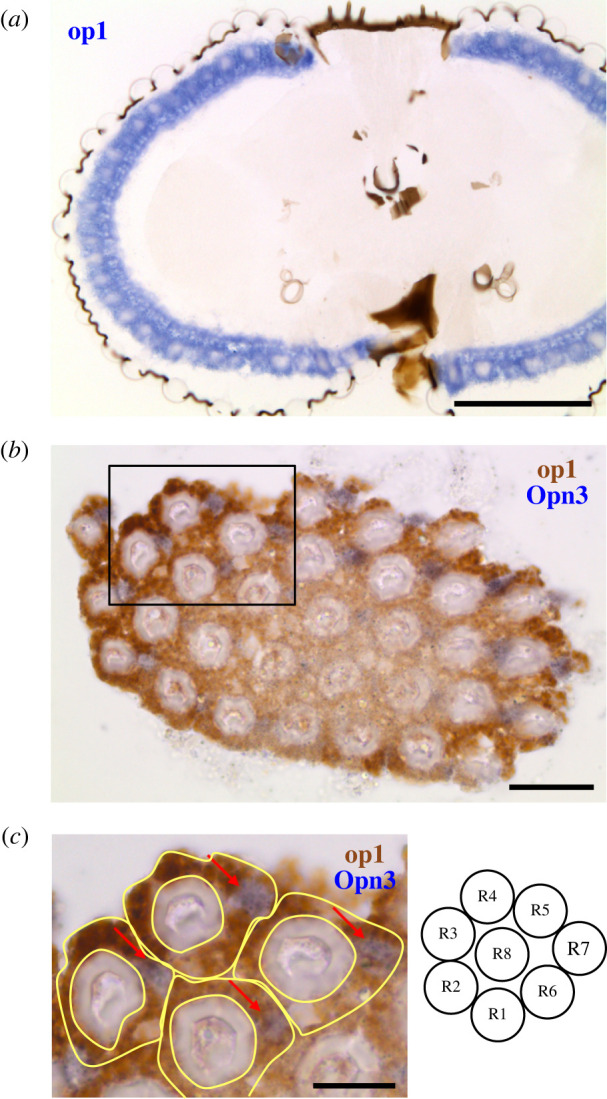
The expression pattern of the LWS visual opsin (Asop1) and MosOpn3 in *A. stephensi* eye. (*a*) *In situ* hybridization analysis of Asop1 in the mosquito eye. The signals derived from Asop1 mRNA (purple) were detected in many ommatidia around the whole eye. (*b*) Double *in situ* hybridization analysis of Asop1 and MosOpn3 (Asop12) in the transverse section of the mosquito eye. Expressions of Asop1 (brown) and MosOpn3 (purple) are visualized. (*c*) The enlarged image from the square in (*b*). The yellow traces indicate the landmarks of each ommatidium containing R1–R7. Judging from the schematic drawing of the transverse view of mosquito ommatidium modified from the previous report [32], Asop1 and MosOpn3 are exclusively expressed in R1–R6 and R7 (arrows), respectively. The scale bars represent 100 μm in (*a*), 20 μm in (*b*) and 10 μm in (*c*).

**Figure 4.**
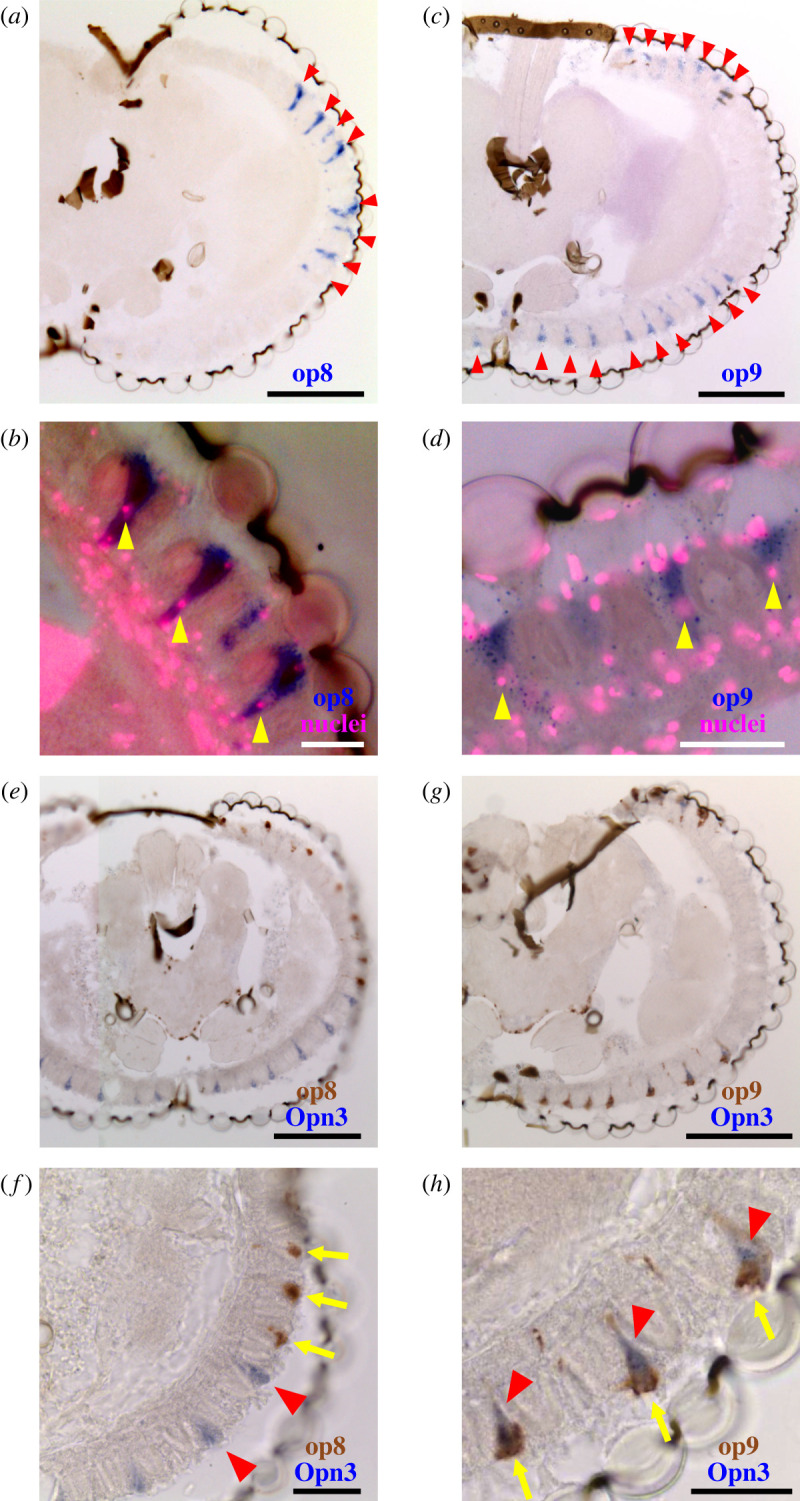
The expression pattern of the UVS (Asop8), the SWS (Asop9) visual opsins and MosOpn3 in the *A. stephensi* eye. (*a,c*) *In situ* hybridization analysis of Asop8 and Asop9 in the mosquito eye. The signals derived from Asop8 ((*a*), arrowheads) and Asop9 ((*c*), arrowheads) mRNAs were detected in a specific kind of photoreceptor cells in dorsal and ventral ommatidia and lateral ommatidia, respectively. (*b,d*) Visualization of cell nuclei using the Hoechst stain (magenta) in the eye, in which Asop8-expressing cells (*b*) or Asop9-expressing cells (*d*) were stained by *in situ* hybridization. The location of nuclei (arrowheads) indicate that Asop8 and Asop9 are expressed in R7 of dorsal and ventral ommatidia and lateral ommatidia, respectively. (*e–h*) Double *in situ* hybridization analysis of Asop8 and MosOpn3 (Asop12) (*e,f*) and Asop9 and MosOpn3 (*g,h*). (*f*) An enlarged image of (*e*), in which expressions of Asop8 (brown) and MosOpn3 (purple) are visualized, indicate the mutually exclusive expression of Asop8 and MosOpn3 in different R7. (*h*) An enlarged image of (*g*), in which expressions of Asop9 (brown) and MosOpn3 (purple) were visualized, indicates co-localization of Asop9 and MosOpn3 in the same R7 of dorsal and ventral ommatidia. Note that signals derived from Asop9 (arrows) and MosOpn3 (arrowheads) mRNAs are distributed differently in R7. The scale bars represent 100 μm in (*a,c,e,g*) and 30 μm in (*b,d,f,h*).

We next compared the relationships between the expression of MosOpn3 with those of canonical visual opsins, which belong to the Gq-coupled opsin group. We focused on Asop1, Asop8 and Asop9 as representatives of insect long-wavelength-sensitive (LWS), ultraviolet-sensitive (UVS) and short-wavelength-sensitive (SWS) visual opsin subgroups, respectively [34,35] (figure 2) to examine their expression patterns by *in situ* hybridization analysis. We found that Asop1 (LWS visual opsin) is expressed in many ommatidia around the whole eye, including dorsal region (figure 3*a*). In the ommatidia, the expression of Asop1 was observed in multiple photoreceptor cells, similar to the case of fruit fly Rh1, in which it is expressed in R1 to R6 among eight photoreceptor cells [36]. Then, we compared the expression pattern of the Asop1 and MosOpn3 by double *in situ* hybridization analysis. In the transverse section of ommatidia, the Asop1-expressing cells form a ring composed of six photoreceptor cells, in which MosOpn3-expressing R7 was not included, showing the mutually exclusive expression of Asop1 and MosOpn3 as well as expression of Asop1 in R1–R6 (figure 3*b,c*). On the other hand, *in situ* hybridization analysis of Asop8 (UVS visual opsin) and Asop9 (SWS visual opsin), together with visualization of cell nuclei revealed that the Asop8 is expressed in R7 of lateral ommatidia (figure 4*a,b*) and the Asop9 is expressed in R7 of dorsal and ventral ommatidia (figure 4*c,d*). The expression of Asop8 in R7 of lateral ommatidia is consistent with the case of *A. gambiae* revealed by immunohistochemical analysis [32]. In *A. gambiae*, Agop2, a member of LWS opsins and closely related to Asop1 [37], is expressed in R7 of dorsal and ventral ommatidia, whereas in *A. stephensi*, the Asop1 is expressed in R1–R6 of ommatidia around the whole eye (figure 3). In *Aedes aegypti*, Aaop9 is expressed in all R7 and a subset of R8, showing the variation among mosquito species [38].

Then, we compared the expression patterns between MosOpn3 and these visual opsins to understand contributions of MosOpn3 to the vision. Double *in situ* hybridization analysis clearly showed that the Asop8 and MosOpn3 are exclusively expressed in different cells (figure 4*e,f*), and the Asop9 and MosOpn3 are expressed in the same cells, indicating co-localization of the Asop9 and MosOpn3 in R7 of the dorsal and ventral ommatidia (figure 4*g,h*).

### (c) Spectroscopic characteristics of the Asop9 expressed in MosOpn3-expressing photoreceptor cells

**Figure 5.**
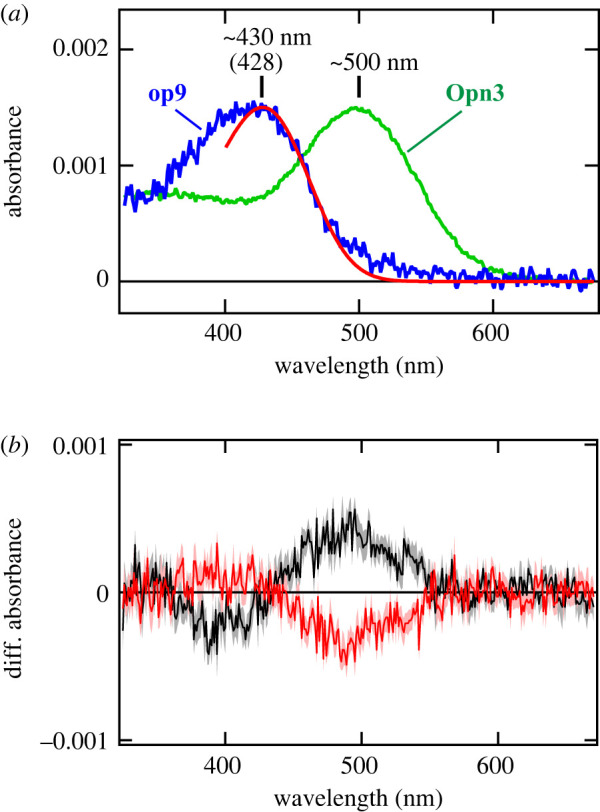
Spectroscopic characteristics of the mosquito blue-sensitive visual opsin (Asop9). (*a*) The absorption spectrum of Asop9-based pigment (blue), fitted with the rhodopsin-nomogram (red) [39] to estimate that the Asop9 forms a blue-sensitive pigment, having an absorption maximum at approximately 430 nm. The absorption spectrum of MosOpn3 having an absorption maximum at approximately 500 nm was also shown (green) [21]. (*b*) The difference absorption spectra, showing the spectral changes of the blue-sensitive Asop9-based pigment caused by irradiation with blue light (black) and subsequent yellow light (red). The photoresponses indicate bistability of Asop9, like other insect visual opsins and MosOpn3. Error bars (in light red and grey around the averaged values shown in dark red and black, respectively) indicate s.e. (*n* = 5). Note that Asop9-based pigment was reconstituted with 11-*cis* form of A1 retinal.

We then experimentally determined the absorption spectrum of Asop9, a SWS visual opsin deduced from the phylogenetic classification (figure 2). We expressed the Asop9 in mammalian cultured cells and successfully obtained the purified Asop9-based pigment reconstituted with A1 retinal as the chromophore. Spectroscopic analysis of the Asop9-based pigment revealed that Asop9 forms the blue-sensitive pigment having an absorption maximum at approximately 430 nm (figure 5*a*). The result is consistent with the case of *Aedes aegypti* Aaop9, which was revealed to have an optimal response to 400–450 nm light by the electroretinogram analysis of transgenic *Drosophila* [38]. Blue light irradiation of the Asop9-based pigment resulted in increase and decrease of the absorbance around 500 nm and around 400 nm, respectively (figure 5*b*). The spectral change by the light irradiation is explained by the conversion of the dark state to the photoproduct having red-shifted absorption spectrum. In addition, subsequent yellow light irradiation caused the opposite reaction, which indicates the reverse conversion of the photoproduct to the dark state. These are typical photoreactions of bistable opsins whose photoproducts also have an absorption maximum in the visible region, showing that Asop9 is a bistable opsin, like other insect visual opsins and MosOpn3.

## 4. Discussions

Molecular basis of insect vision has been investigated mainly in the fruit fly, and the Gq-mediated phototransduction has been believed to be the sole visual phototransduction in insects; G*α*q subunit activates PLC*β*, and depletion of PIP2 caused by PLC*β* leads to activation of TRP/TRPL channels and then to photoreceptor cell depolarizations [2,40]. That was reasonable because the fruit fly only has Gq-coupled opsins. In this paper, we focused on an *Anopheles* mosquito, which has an opsin, MosOpn3 (Asop12), previously reported to form a green-sensitive Gi/Go-coupled pigment, in addition to canonical Gq-coupled opsins [21]. We found that MosOpn3 is expressed in the mosquito eye, specifically in R7 photoreceptors among eight photoreceptors of dorsal and ventral ommatidia (figure 1), which is to our knowledge the first report in any animal of Opn3 expression in visual photoreceptor cells. We also showed that the blue-sensitive visual opsin (Asop9) is co-expressed with MosOpn3 in R7 of dorsal and ventral ommatidia, whereas in R7 of lateral ommatidia, the UVS visual opsin (Asop8) is expressed but MosOpn3 is not, suggesting functional division of the two regions in the mosquito eye (figures 4 and 5*a*). Understanding the region-dependent variation in physiology would provide a clue to the physiological meaning of the co-localization. In the fruit fly, R7 of all ommatidia express UVS opsin, Rh3 or Rh4 and underlie colour vision together with R8 [41,42]. Colour vision of mosquito was recently reported but an involvement of R7 in the mosquito colour vision is still unknown [43]. Further investigations would be needed to clarify whether the mosquito R7 photoreceptors underlie colour vision like in fruit flies or alternatively, are involved in mosquito-specific unknown physiologies.

Coexistence of two opsins has been reported in several insects such as fruit fly, butterfly and red flour beetle [44–46]. In those cases, however, both opsins are canonical insect visual opsins, members of the Gq-coupled opsin group. Outside insects, coexistence of two opsins with different G protein selectivity has been known in the photoreceptive portion of a single photoreceptor cell of lizard parietal eyes and teleost pineal organs [47–49] and photoreceptor cells of some invertebrates [50–52]. In the well-studied pineal-related organs of lower vertebrates, the UVS opsin (parapinopsin) or the blue-sensitive opsin (pinopsin) and the green-sensitive opsin (parietopsin) light-dependently activate Gt to decrease and Go to increase the intracellular cGMP level, respectively. The antagonistic response generates colour opponency, which could allow for wavelength discrimination by detecting the ratio between UV/blue and green [47,49]. If MosOpn3 and Asop9 are co-localized in the photoreceptive portion, rhabdomere of R7 of dorsal and ventral ommatidia, wavelength discrimination with a single photoreceptor could be possible by an antagonistic regulation involving the Gq-mediated signalling and the Gi/Go-mediated cAMP/cGMP signalling. Alternatively, according to a recent report that Gi-mediated signalling enhances the Gq-mediated signalling [53], the light response based on the blue-sensitive Asop9 could be enhanced in the blue to green region by the green-sensitive MosOpn3. These ideas, however, are plausible when both MosOpn3 and Asop9 are co-localized in the same rhabdomere but direct evidence that shows co-localizations of two opsins in the R7 rhabdomere still remains to be found.

Interestingly, our double *in situ* hybridization analysis showed a different distribution of their mRNA; Asop9 mRNA is located near the lens and rhabdomere and MosOpn3 mRNA is located apart from them and relatively close to the nerve terminal (figure 4*h*). Judging from the previous findings that the distribution of mRNA is correlated with that of the protein [54,55], the different mRNA distribution could suggest more likely that Asop9 is located at rhabdomeres, but MosOpn3 is not and rather at the cytoplasm or terminal of R7. The idea is intriguing because at *Drosophila* photoreceptor synapses, histamine H3 receptors—which are Gi-coupled—receive a neurotransmitter histamine to generate feedback loops [56], indicating the presence of G*α*i subunits at photoreceptor synapses. Moreover, in *Drosophila* R7, the Gi-mediated signalling triggered by activation of histamine H3 receptors is involved in processing colour opponency at the photoreceptor level [42]. The Gi-mediated signal transduction at the synapses could be light-dependently activated by MosOpn3, resulting in the contribution of MosOpn3 to the light response processed by R7. Further investigation of the cellular localization of MosOpn3 is required to precisely discuss its function.

Many insects possess Opn3 homologues in addition to canonical Gq-coupled opsins; in other words, ‘Opn3-less’ insects, including the fruit fly, are rare (figure 2). Therefore, the co-expression of Gq and Gi/Go-coupled opsins might occur in photoreceptors of other insects, potentially modifying their visual function.

Thousands of opsins identified from varied animals are categorized as c-opsins (also called ciliary opsins) or r-opsins (also called rhabdomeric opsins). The former trigger cyclic nucleotide phototransductions via Gt, Gi/o or Gs in ciliary photoreceptor cells, and the latter trigger phosphoinositol phototransductions via Gq in rhabdomeric photoreceptor cells [13,17,57]. Opn3 is assigned to c-opsin based on the evidence that annelid worm Opn3 is expressed in ciliary photoreceptor cells in the brain [17], and several Opn3 s including MosOpn3 trigger cyclic nucleotide phototransductions at least *in vitro* [21,58]. Together with the fact that insect visual cells are rhabdomeric-type, current findings that the Opn3 homologue is expressed in visual cells in the mosquito are exceptional, indicating the plasticity of the relationship between opsin and photoreceptor cell type.

## Data Availability

DNA sequences reported in this paper are available in public databases under the INSDC accession nos. LC710138–LC710140.
